# Systematic review of economic evaluations of exercise and physiotherapy for patients treated for breast cancer

**DOI:** 10.1007/s10549-019-05235-7

**Published:** 2019-04-17

**Authors:** Kamran Ahmad Khan, Bruno Mazuquin, Alastair Canaway, Stavros Petrou, Julie Bruce

**Affiliations:** Warwick Clinical Trials Unit, Warwick Medical School, University of Warwick, Gibbet Hill Road, Coventry, CV4 7AL UK

**Keywords:** Exercise, Rehabilitation, Cost-effectiveness, Systematic review, Economic evaluation

## Abstract

**Purpose:**

Treatments for breast cancer can lead to chronic musculoskeletal problems. This study aimed to systematically review the evidence surrounding the cost-effectiveness of exercise and physiotherapy interventions aimed at reducing the risk of physical symptoms and functional limitations due to breast cancer treatment.

**Methods:**

A systematic review of the cost-effectiveness of exercise and physiotherapy interventions during and following treatment for breast cancer was undertaken according to PRISMA guidelines. Literature searches were carried out in Ovid MEDLINE, Ovid Embase, Web of Science, EconLit, CINAHL, PsycINFO, Scopus and the Cochrane Library. Cost-effectiveness evidence was summarised in a descriptive manner and studies were assessed using quality appraisal tools. The review protocol was registered on PROSPERO.

**Results:**

A total of 7783 articles were identified and seven were included in the final review. Five studies undertook trial-based economic evaluations, whereas two studies conducted economic evaluation based on decision models. One study was a cost-effectiveness analysis (CEA), three undertook stand-alone cost–utility analyses (CUA) and three studies were combined CEAs and CUAs. Three studies reported favourable cost-effectiveness results for different exercise or physiotherapy interventions. In contrast, four studies found that exercise and physiotherapy interventions were not cost-effective on the basis of quality-adjusted life year outcomes.

**Conclusions:**

The evidence surrounding the cost-effectiveness of exercise and physiotherapy interventions for the treatment of breast cancer remains sparse with contrasting conclusions. Future research should particularly aim to broaden the evidence base by disentangling the contributing effects of frequency, intensity, time and type of exercise and physiotherapy interventions on cost-effectiveness outcomes.

**Electronic supplementary material:**

The online version of this article (10.1007/s10549-019-05235-7) contains supplementary material, which is available to authorized users.

## Introduction

Breast cancer is the most common form of cancer amongst women in the United Kingdom (UK) with approximately 50,000 new cases diagnosed each year [1]. Most women diagnosed with breast cancer have surgery to the breast and axilla, with many also requiring radiotherapy and chemotherapy [2, 3]. These treatments can affect the muscles, nerves and lymphatic vessels in the shoulder and upper body, resulting in musculoskeletal problems such as limited range of motion, weakness, persistent pain, altered sensations and lymphoedema [4, 5]. Studies suggest that between 10 and 64% of women have symptoms in their arm or shoulder up to 3 years after treatment [6]. These persistent symptoms can delay recovery, limit daily activities and impair health-related quality of life (HRQoL). It is important that the UK National Health Service (NHS) and health systems in other countries provide adequate care for women to ensure recovery and return to usual activities after cancer treatment.

Exercise interventions may alleviate the side-effects of cancer treatment with several systematic reviews of literature suggesting they may be clinically effective [7–10]. McNeely et al. [8]., for example, reported that exercise improves HRQoL and physical capacity and reduces fatigue in breast cancer patients. Furthermore, physical activity can improve cardiorespiratory capacity and well-being in cancer patients [9].

Although exercise interventions have been shown to be clinically effective in several studies, information on their cost-effectiveness is sparse. Two systematic reviews, one investigating the cost-effectiveness of exercise-based interventions in the treatment of various chronic diseases [11] and the other the cost-effectiveness of cancer rehabilitation [10], identified only two economic evaluations of physical activity interventions for breast cancer patients [12, 13]. The first study reported that a home-based self-managed physiotherapy intervention and a supervised group-based exercise intervention with psychosocial support were more cost-effective than usual care [12]. In contrast, the second study concluded that a home-based self-managed exercise intervention was not cost-effective compared to an active control consisting of flexibility and relaxation activities after breast cancer surgery [13].

Given the limited resources in public health systems, healthcare interventions should seek to maximise health benefits or broader measures of social welfare with the resources available. To achieve efficient resource allocation, only methods of proven cost-effectiveness should be adopted for routine use in the NHS and other publicly funded health systems [14–17]. Therefore, the aim of this study was to systematically review evidence on the cost-effectiveness of exercise and physiotherapy interventions for the treatment of breast cancer to inform policy decisions in this clinical area.

## Methods

A systematic review of the literature, following Preferred Reporting Items for Systematic Review and Meta-Analyses (PRISMA) guidelines [18], on the cost-effectiveness of exercise and physiotherapy during and following treatment for breast cancer was undertaken. Literature searches were carried out in Ovid MEDLINE, PubMed, Ovid Embase, Web of Science, EconLit, CINAHL, PsycINFO, Scopus and The Cochrane Library (including the NHS Economic Evaluation Database (NHSEED) electronic databases with time horizons covering inception of the databases to 24 September 2018. Biomedical databases were searched using various combinations of keywords and medical subject headings (MeSH) based on terms relevant to breast cancer, physiotherapy, exercise or physical activity and economic evaluation. Further details on the search strategies applied to each database are available in the Online Resource 1. Searches were not limited by date of publication or language. The review protocol was registered on PROSPERO (CRD42018108978).

### Selection criteria

Economic evaluations of exercise and physiotherapy interventions for breast cancer patients were considered. Eligible types of economic evaluations included cost-effectiveness analyses, cost–benefit analyses, cost–utility analyses, cost consequences analyses and cost-minimisation analyses. Each study was required to have reported both costs and consequences and compared an experimental intervention to at least one other intervention or control. Participants included in the selected studies were adults with a confirmed breast cancer diagnosis who were undergoing or had received treatment, including any surgical removal of breast tumour, e.g. mastectomy (simple, modified or radical), local wide excision or lumpectomy and/or axillary surgery (lymph node dissection/clearance or sentinel lymph node biopsy (SNB/SNLB) or dissection. An exercise or physiotherapy intervention was defined as one that included an exercise intervention delivered and supported or unsupported by a physiotherapist or other health professional. Comparators included usual care/control, different types of exercises or no exercise. Descriptions of usual care/control were extracted from primary reports. Outcome measures included measures of cost-effectiveness, e.g. an incremental cost-effectiveness ratio (ICER) or a measure of net monetary benefit (NMB). All identified titles and abstracts were screened independently by two authors (KK and BM) and, where relevant, full-text articles were obtained and assessed against the study inclusion criteria. Disagreements at each stage (title and abstract stage, full report stage) were resolved by discussion or referred to a third author (SP) for final assessment.

### Data extraction

Data extraction was carried out by one reviewer (KK) and checked by a second reviewer (BM). Any disagreements were resolved through discussion or through a final assessment by a third reviewer (SP). Data were extracted using a standardised form. Extracted data items included author(s), year of publication, country and setting, patient characteristics, intervention and comparator details, main analytical approaches (e.g. patient-level analysis or decision-analytic modelling) and the primary outcome(s) specified for the economic analysis. In addition, details of estimation and adjustment for HRQoL, key assumptions made in the base case or tested in sensitivity analyses, direct costs (medical and non-medical) and productivity costs estimated, estimates of cost-effectiveness and approaches to quantifying uncertainty (e.g. decision uncertainty to address uncertainty around the value of the cost-effectiveness threshold, probabilistic sensitivity analysis to address uncertainty surrounding the value of parameter inputs) were extracted.

### Quality assessment

The quality of reporting by the economic evaluations was assessed using the Consolidated Health Economic Evaluation Reporting Standards (CHEERS) checklist [19]. The quality of each economic evaluation was scored using CHEERS criteria, which allows overall scores from 0 to 24 (24 representing the best score possible). In addition, the methodological quality of any randomised controlled trial underpinning an economic evaluation was assessed using the Cochrane Collaboration’s risk of bias tool [20]. The risk of bias domains included random sequence generation, allocation concealment, blinding of participants, blinding of personnel, blinding of outcome assessment, incomplete outcome data and selective outcome reporting. Each domain was classified as of low, high or unclear risk [20]. Where studies failed to report an item, it was classified as unclear.

### Analysis

Cost data extracted from studies were inflated, where necessary, to 2016 prices using the relevant country-specific Gross Domestic Product (GDP) deflator index, and subsequently converted, where necessary, from their respective currencies into US dollars using purchasing power parities supplied by the Organisation for Economic Co-operation and Development (OECD) [21]. For studies that failed to report their currency price dates, it was assumed that the costs used in the valuation process applied to the financial year prior to the publication of the study.

Methodological variations between studies, including variations in underpinning health care practices across jurisdictions and variations in the relative prices of labour and capital inputs across jurisdictions, prevented a pooling of economic data akin to meta-analyses performed on clinical effectiveness estimates. Rather, cost-effectiveness estimates and broader economic outcomes are presented in a descriptive manner according to broad economic design.

## Results

### Search results

**Fig. 1:**
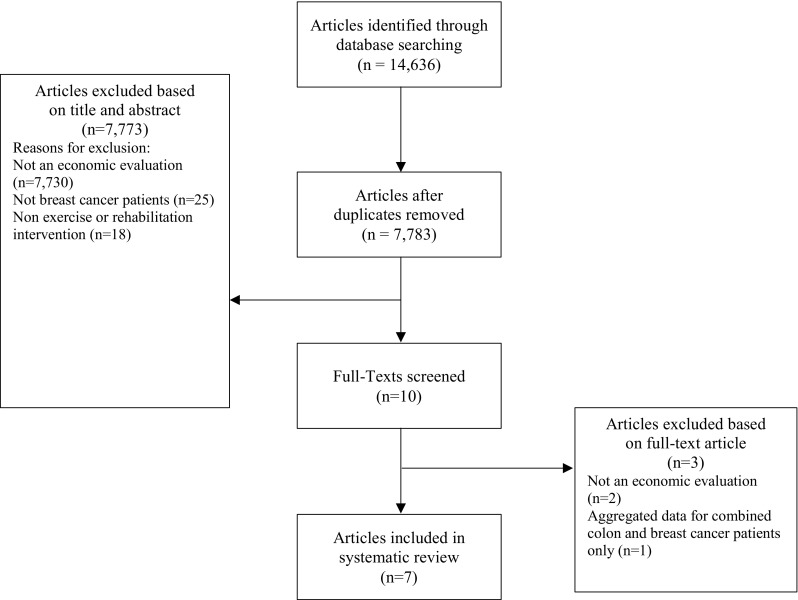
PRISMA flow diagram

In total, 14,636 records were identified from the bibliographic searches. After removing 6853 duplicates, 7783 titles and abstracts were reviewed and 7773 articles were subsequently excluded at the title and abstract screening stage (Fig. 1). Common reasons for exclusion were that the studies were not economic evaluations, the population did not include breast cancer patients or the intervention was not exercise or physiotherapy based. Ten articles fulfilled screening criteria and were retrieved for full-text analysis; of these, seven fulfilled the study inclusion criteria. Two studies were excluded at the full report stage because they were not economic evaluations [22, 23], whilst a third study by Kampschoff and colleagues [24] presented only aggregated results across breast and colon cancer patients and it was not possible to obtain data only for breast cancer (Fig. 1). Of the seven included studies, one study by Perrier and colleagues [25] was reported as a conference poster and further details were obtained directly from the authors.

### Study characteristics

Descriptive information (study design, patient characteristics, interventions, outcomes) pertaining to each included study is presented in Table 1. Classified by country of origin, three studies were conducted in the Netherlands, three conducted in Australia and one in France. Five studies carried out trial-based economic evaluations [13, 25–28], whereas two studies conducted economic evaluation based on decision models [12, 29]. A decision model theoretically allows for the extrapolation of costs and effects beyond the time horizon of trial data, can reflect all appropriate evidence, can compare all relevant options and can make head-to-head comparisons of alternative competing interventions when relevant trials do not exist [30, 31]. The study by Gordon et al. [12] made head-to-head comparisons of alternative competing interventions without trial-based data, whereas Mewes et al. [29] used decision modelling to extrapolate costs and effects beyond the time horizon of the trial data used [32].

**Table 1:** Description of methods of included economic evaluations

Authors (country)		Gordon et al. [12] (Australia)	Gordon et al. [26] (Australia)	Haines et al. [13] (Australia)	May et al. [27] (The Netherlands)	Mewes et al. [29] (The Netherlands)	Perrier et al. [25] (France)	Van Waart et al. [28] (The Netherlands)
Year		2005	2017	2010	2017	2013	2016	2018
Study design		Model based economic evaluation Study design is observational for both DAART and STRETCH	RCT with economic evaluation	RCT with economic evaluation	RCT with economic evaluation	Model based economic evaluation Hypothetical cohort of 1000	RCT with economic evaluation	RCT with economic evaluation
Period of intervention		STRETCH—8 weeks, up to 12 months follow-up DART—6 weeks, up to 12 months follow-up	8 months, up to 12 months follow-up	6 months, up to 12 months follow-up	18 weeks, up to 9 months follow-up	12 weeks, up to 6 months follow-up	6 months	1–4 months, 6 months follow-up
Analytic horizon		12 months	12 months	6 months	9 months	5 years	6 months	Less than 12 months
Setting		Community	Hospital and community	Hospital and community	Outpatient clinics		Community	Community
Population		Women with breast cancer, aged 25–74	Women with breast cancer aged 20–69, resident in Brisbane area	Women with newly diagnosed breast cancer undergoing adjuvant therapy	Histological diagnosis of breast cancer < 6 weeks pre- recruitment; stage M0; scheduled for chemotherapy; Age 25–75 years; no cancer treatment in 5 years preceding recruitment	Female breast cancer patients; aged < 50 years, premenopausal, had undergone adjuvant chemotherapy and/or hormonal therapy, had experienced a treatment-induced menopause and who reported at least a minimal level of menopausal symptoms	Breast cancer patients receiving first-line adjuvant chemotherapy	Patients scheduled for adjuvant chemotherapy for breast cancer at one of the 12 participating hospitals in wider Amsterdam region of the Netherlands between 2010 and 2012
Sample size	Study Group	DAART *n* = 36 STRETCH *n* = 31	Treatment *n* = 134	Intervention *n* = 46	Intervention *n* = 87		Intervention (*n* = 41)	OnTrack (*n* = 76) OncoMove (*n* = 77)
	Control group	Usual care (*n* = 208)	Usual care *n* = 60	Control *n* = 43	Control *n* = 78		Control (*n* = 19)	Control (*n* = 77)
Age, mean (SD)		DAART 59 (10.7) STRETCH 54 (11.3) Usual care 55 (10.3)	52 (8)	Intervention 55.9 (10.5) Control 54.2 (11.5)	Intervention 50.0 (7.9) Control 49.4 (7.6)	Age < 50 years	18–75 years	OnTrack 49.9 (8.4) OncoMove 50.5 (10.1) Usual Care 51.6 (8.8)
Intervention		DAART—early home-based physiotherapy intervention. Main components: Recovery of shoulder range of movement (SROM), Education, Tailored exercise prescription for self-management STRETCH—a group-based exercise and psychosocial intervention. Main components: Recovery of SROM, Education, Discussion of psychosocial issues	8-month exercise programme for women after surgery for primary breast cancer. Intervention delivered through either face‐to‐face home delivery or by telephone over an 8‐month period starting 6 weeks after breast surgery. The intervention involved 16 scheduled sessions	Participants received a multimedia instructional package with equipment. A programme combining a range of exercise approaches, with balance and shoulder mobility components Included progression	Supervised 1-h aerobic and resistance exercise (twice per week for 18 weeks). The programmeme was individualised to the patient’s personal preferences and fitness level. The 1-h exercise classes included a warming up (5 min), aerobic and muscle strength training (50 min) and a cooling down (5 min). Recommended to be physically active for at least 30 min a day on at least three other days	Physical exercise intervention consisting of a 12-week home-based exercise programme, individually tailored during an intake session with a physiotherapist	6-month supervised physical activity programme of indoor and outdoor group sessions in addition to usual dietary advice	OnTrack: moderate-to-high intensity, combined resistance and aerobic exercise programme, supervised by specially trained physiotherapists. Twice a week, six large muscle groups are trained, followed by 30 min of aerobic exercises OncoMove: home-based, low-intensity, individualised, self-managed physical activity programme, with the addition of behavioural reinforcement. Specially trained nurses encourage participants at each chemotherapy cycle to engage in 30 min of physical activity per day, 5 days a week
Control		Usual care, a population-based sample, representative of women with breast cancer in the same geographic area	Usual care group received no regular or formal advice outside of routine health care contacts	Active (sham intervention) control of flexibility and relaxation activities Video and supporting material looked but actual exercises described differed. No progression	Usual care—maintain habitual physical activity pattern up to week 18. Thereafter, offered routine exercise programmes after cancer treatment	usual care, waiting list control	Dietary advice only	Varied according to hospital guidelines and preferences but did not involve routine exercise
Primary health outcome of the study		DAART study outcomes: shoulder ROM, arm circumference, function, pain, STRETCH: shoulder ROM	QoL—FACT-B + 4	HRQoL—EQ-5D, EORTC C30	Fatigue—MFI	Endocrine symptoms—FACT-ES	Adherence to the intervention	Cardiorespiratory fitness—endurance and heart rate at the end of an incremental bicycle ergometer test

*DAART* Domiciliary Allied Health and Acute Care Rehabilitation Team, *EORTC C30* European Organisation for Research and Treatment of Cancer Core questionnaire, *EQ*-*5D* EuroQol 5 dimensions, *FACT*-*B* +*4* Functional Assessment of Cancer Therapy-Breast, *FACT*-*ES* Functional Assessment of Cancer Therapy Questionnaire Endocrine Scale, *MFI* multidimensional fatigue inventory and fatigue quality list, *NA* not applicable, *QoL* quality of life, *RCT* randomised control trial, *STRETCH* Strength Through Recreation Exercise Togetherness Care Health, *SROM* shoulder range of movement

### Interventions and outcomes

The type of physiotherapy and exercise interventions evaluated by the studies included home-based self-managed exercises [13, 28, 29], home-based self-managed and supervised physiotherapy [12], home-based supervised exercises with different delivery methods (face-to-face or over-the-telephone) [26] and group-based supervised exercise programmes [12, 25, 27, 28]. The physiotherapy and exercise interventions targeted a range of health and fitness goals including strength and flexibility, balance, endurance and overall fitness (Table 1). The range of control interventions included usual care [12, 26, 27, 29], a sham intervention (active control of flexibility and relaxation activities) [13], dietary advice [25] and usual care with no routine exercise [28]. For the five studies using trial data, the primary outcome measures included the self-report Functional Assessment of Cancer Therapy—Breast Cancer version 4 (FACT-B + 4) [26], EuroQol 5-dimension questionnaire 3 level (EQ-5D-3L) [13], European Organisation for Research and Treatment of Cancer core quality of life questionnaire (EORTC QLC-C30) [13], cardiorespiratory fitness [28] and the multidimensional fatigue inventory (MFI) and fatigue quality list (FQI) [27].

### Economic evaluations

Information relating to the characteristics and economic outcomes of the economic evaluations is presented in Table 2. All economic evaluations were published between 2005 and 2018. One study was cost-effectiveness analysis (CEA) [25], three were stand-alone cost–utility analyses (CUAs) [13, 27, 29] and three were combined CEAs and CUAs [12, 26, 28]. Four studies adopted a societal perspective [12, 13, 27, 28], whilst one adopted a health care system perspective [29], one a private and service provider perspective [26] and one a national insurance perspective [25]. The mean total costs per patient for delivering group exercise interventions ranged from AUS$342 (US$327, 2016 prices) for a home-based physiotherapy intervention [12] to €31,133 (US$38,819, 2016 prices) for a home-based, low-intensity, individualised, self-managed physical activity programme with the addition of behavioural reinforcement [28]. The primary measure(s) of health consequence included in the seven economic evaluations fell into the following categories: number of rehabilitated cases and quality-adjusted life-years (QALYs) [12, 26]; change in body mass index (BMI) and cardiorespiratory fitness [25]; fatigue and QALYs [28] and QALYs alone [13, 27, 29]. QALYs were derived from the EQ-5D-3L measure in four studies [13, 26–28], whilst in one study [12], QALYs were generated by multiplying period of life by utility scores obtained using a single-item linear analogue scale entitled the Subjective Health Estimation (SHE) scale which had been developed and validated by the International Breast Cancer Study Group [33]. A further study used a mapping algorithm to estimate EQ-5D utility scores from the short-form six dimension health index (SF-6D) and then used those values to calculate QALYs [29].

**Table 2:** Outcomes of included economic evaluations

Authors (Country)	Gordon et al. [12] (Australia)	Gordon et al. [26] (Australia)	Haines et al. [13] (Australia)	May et al. [27] (The Netherlands)	Mewes et al. [29] (The Netherlands)	Perrier et al. [25] (France)	Van Waart et al. [28] (The Netherlands)
Type of study	CEA & CUA, Model based, Markov model 12 months	CEA & CUA Trial based	CUA Trial based	CUA Trial based	CUA Model based Markov model 5 years	CEA Trial based	CEA & CUA Trial based
Perspective	Societal	Service provider, private	Societal	Societal	healthcare system perspective	French national insurance perspective	Societal
Costs (Currency, price date, types of costs, sources of cost data, valuation of costs. discount rate)	2004 AUS $, Direct and indirect, Literature, national tariffs No discounting Average cost DAART AUS $342 STRETCH AUS $1038 Usual Care (UC) AUS $189 Incremental cost versus UC DAART AUS $133 STRETCH AUS $941	2014 AUS $, Intervention, out of pocket, Trial records, invoices No discounting Mean costs Service provider AUS $967 Private AUS $838 Usual care AUS $20	2006 AUS $, Intervention, Direct health and productivity Trial data, Market prices, Australian DRG cost weights, mean wage rates No discounting Total costs Median (IQR), mean Intervention AUS $3864 (2450, 10,076), 10,082 Control AUS $3594 (2316, 7992), 3819	2011 Euros €, Direct and indirect, Trial data, own cost price calculations, No discounting Total societal costs, Mean (SD) Intervention €25,105 (10,403) Control €22,215 (8652)	2011 Euros €, Intervention costs, Health care costs, Medication, Literature, national tariffs Discount rate 4% Total cost Intervention €2983 Control €2798	2012 Euros €, Intervention, total, Trial records, No discounting Total costs, mean (SD) Intervention €15,776 (9772) Control €18,475 (14,612)	2017 Euros €, Intervention, Direct Health care, Absenteeism, Unpaid productivity Trial data, National tariffs No discounting Total costs, mean (SE) OnTrack €29,589 (1615) OncoMove €31,133 (2236) Usual Care €28,714 (1984)
Effects (type of effects, sources of QALYs, discount rate)	Rehabilitated cases *n* (%) QALYs (Subjective Health Estimation (SHE) scale) No discounting Rehabilitated cases *n* (%) DAART 14 (45%) STRETCH 12 (48%) Usual care 99 (52%) Utility score, mean (SD) DAART 0.77 (0.19) STRETCH 0.79 (0.18) Usual care 0.73 (0.17)	Rehabilitated cases QALYs (EQ‐5D‐3L) No discounting Improvers Intervention 69 Usual care 21 Mean QALYs Intervention 0.846 Usual care 0.837	Utility scores (EQ‐5D‐3L) No discounting Utility score, mean (SD) Intervention 0.80 (0.21) Control 0.83 (0.18)	QALYs (EQ‐5D‐3L) No discounting QALYs total, mean (SD) Intervention 0.569 (0.03) Control 0.560 (0.04)	QALYs (EQ-5D derived/mapped from SF-6D) Discount rate 1.5% Total QALYs Intervention 4.399 Control 4.392	Change in BMI score VO_2_max gained No discounting Change in BMI score Intervention 0.05 Control 0.29 Change in VO_2_max Intervention 0.39 Control − 0.06	QALYs (EQ-5D-3L) General fatigue Physical fatigue No discounting QALYs gained, mean (SE) OnTrack 0.65 (0.01) OncoMove 0.63 (0.02) Usual Care 0.58 (0.02)
Outcomes	Incremental cost per rehab case DAART Dominated by UC STRETCH Dominated by UC ICER QALYs DAART versus UC AUS $1344 STRETCH versus UC AUS $14,478	ICER improvers Service provider AUS $ 2644 Private AUS $ 2282 ICER QALYs Service provider AUS $105,231 Private AUS $90,842	Only 5% probability that the intervention would be both less costly and more effective than the control	Incremental costs €2912 Incremental QALYs 0.01 ICER was €291,200	Incremental costs €185 Incremental QALYs 0.0067 ICER €28,078	ICERS €-11,159 per BMI unit lost €-6030 per estimated aerobic capacity unit gained for VO_2_max Intervention dominates usual care	Incremental cost OnTrack versus UC 1184 OncoMove versus UC 2571 Incremental QALYs OnTrack versus UC 0.04 OncoMove versus UC 0.04 ICERs Improvement in general fatigue OnTrack versus UC 788 OncoMove versus UC 4711 Improvement in physical fatigue OnTrack versus UC 1402 OncoMove versus UC 10,384 QALYs OnTrack versus UC 26,916 OncoMove versus UC 70,052
Sensitivity analyses conducted	A one-way sensitivity analysis was performed for several cost and outcome estimates PSA of cost-effectiveness inputs	One-way sensitivity of QALYs and costs PSA of cost-effectiveness inputs	None	Scenario analysis—Cost-effectiveness from healthcare perspective	One-way sensitivity PSA	PSA	Scenario analysis
Results of sensitivity analyses	The ICERs for the STRETCH and DAART interventions remained robust to nearly all sensitivity analysis, with the exception of varying utility scores to their lower confidence limits when QALYs were the outcome used	Sensitivity analyses indicated that the incremental cost‐effectiveness ratios using QALYs gained were most sensitive when the EQ‐5D-3L utility values were varied within their 95% confidence limits. Other variations in variables tested (e.g. leasing costs) produced negligible changes to the incremental cost-effectiveness ratios. The likelihood of the service provider model being cost-effective was 44.4%, and 46.3% for the private model, at a cost-effectiveness threshold of AUS$50 000 per QALY gained	NA	Similar to results of the baseline analysis	The outcomes were most influenced by (1) the utility values of the “menopausal symptoms” and “reduction in menopausal symptoms” health states, and (2) the duration of the treatment effect, with shorter effect duration resulting in lower cost-effectiveness The outcomes of this study were most sensitive to a reduction of the duration of the treatment effect from 5 to 3 and 1.5 years	Probability that intervention is cost-effective reached 56% for the BMI outcome measure and 69% for the VO2max outcome measure	The probability of cost-effectiveness for both comparators was greater amongst compliant participants
Conclusion	Rehabilitated cases—not cost-effective when rehabilitated cases were used as the outcome for generating the ICER, the usual care group was superior to both STRETCH and DAART interventions When QALYs were used, the DAART group was more effective than both STRETCH and usual care	In this study, the EQ‐5D‐3L was not sensitive to capture the intervention effect, and therefore, QALYs were not entirely appropriate for this context In terms of the numbers of women reporting clinically significant improvements in quality of life, the intervention, using either service model, may be cost‐effective at approximately A$2400 per improver (or A$300 per month)	Not cost-effective Provision of multimodal exercise programmes will improve the short-term health of women undergoing adjuvant therapy for breast cancer but are of questionable economic efficiency	Not cost-effective Probability that the intervention would be cost-effective at 20,000 threshold is 2%	Physical Exercise is a cost-effective strategy for alleviating treatment-induced menopausal symptoms in this population	On the basis of both cost and effectiveness, the study finds potential advantages in using 6-month supervised physical activity programme in addition to the usual dietetic care instead of one dietetic care only	OncoMove is not likely to be cost-effective Depending on the decision-makers’ willingness-to-pay, OnTrack could be considered cost-effective in comparison with UC Both interventions had a low probability of being cost-effective for physical fitness
Quality score^a^	22	20	20	20	22	19	

*CEA* Cost-effectiveness analyses, *CI* confidence interval, *CUA* cost–utility analyses, *DAART* Domiciliary Allied Health and Acute Care Rehabilitation Team, *DRG* Diagnosis-Related Grouping, *EQ*-*5D*-*3L* EuroQol generic health questionnaire 3 level version, *ICER* incremental cost-effectiveness ratio, *IQR* inter quartile range, *PSA* probabilistic sensitivity analyses, *QALYs* quality-adjusted life-years, *SD* standard deviation, *SE* standard error, *STRETCH* Strength Through Recreation Exercise Togetherness Care Health

^a^Quality assessment based on the Consolidated Health Economic Evaluation Reporting Standards (CHEERS) statement [34]

### Quality of studies

**Fig. 2:**
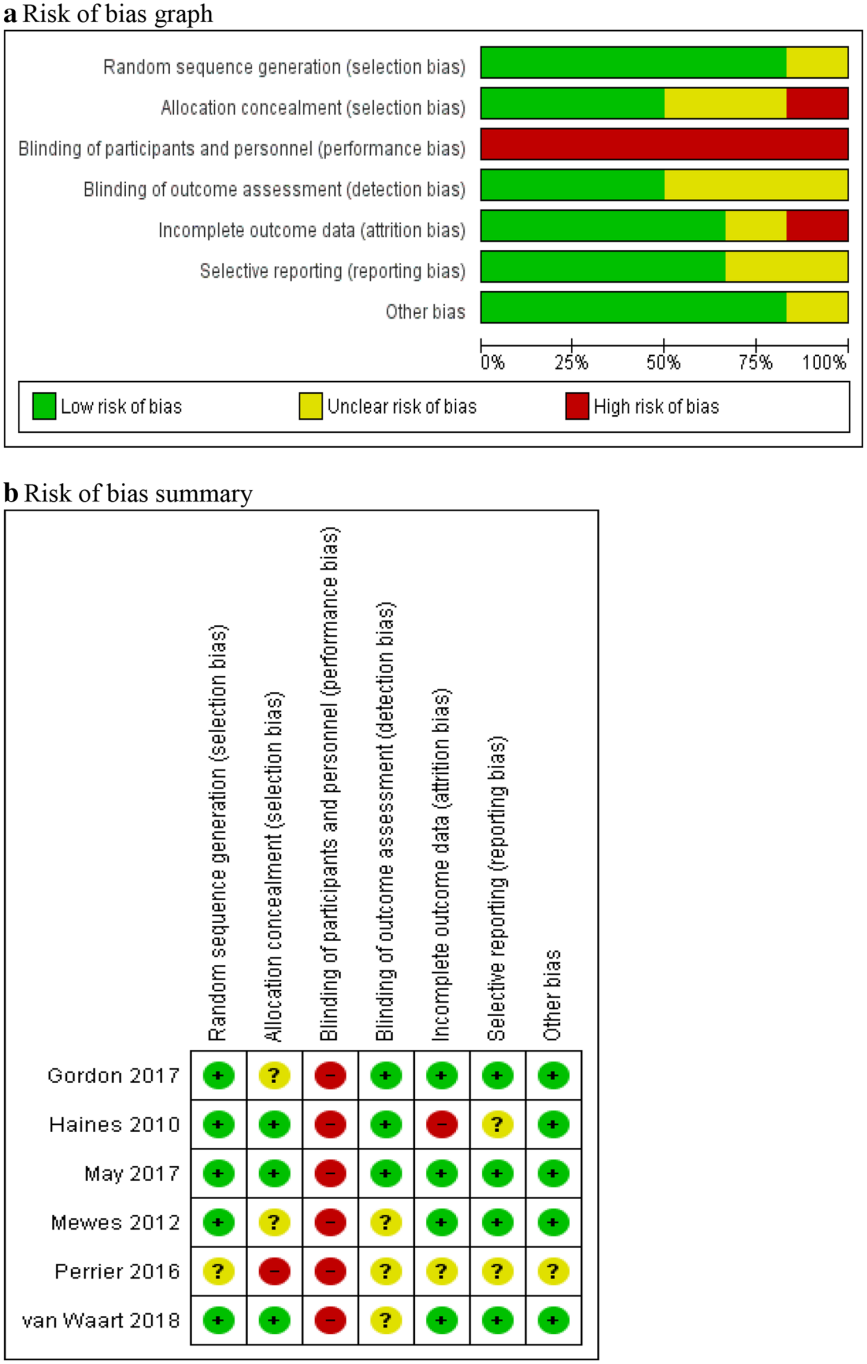
Risk of bias assessment

The methodological quality assessment of the economic evaluations as judged by the CHEERS checklist produced scores ranging from 19 to 22 (Online Resource 2). For risk of bias, although there were five studies that carried out trial-based economic evaluations [13, 25–28], Mewes et al. [29] used a decision model that drew upon data from a trial [32]; therefore, risk of bias results are presented for six studies. The methodological assessment of risk of bias was consistent across studies (Fig. 2, Online Resource 3) with the majority of studies considered at low of risk of bias for the majority of domains. However, all studies were considered at high risk of bias for the domain “blinding of participants and personnel” due to the unconcealed nature of the exercise and physiotherapy interventions.

### Summary of results of economic evaluations

The results pertaining to the cost-effectiveness of the exercise and physiotherapy interventions for breast cancer patients evaluated are summarised in Table 2.

### Cost-effectiveness studies

Only one economic evaluation, a study conducted in France, reported non-QALY based results alone and found that a supervised group-based intervention dominated usual care (i.e. generated improved health outcomes and lower overall costs, on average) with a negative ICER of €-11,159 per decline in BMI unit [25]. The results for cardiorespiratory fitness also showed that the intervention dominated usual care with a negative ICER of €-6030 per estimated aerobic capacity unit gained for VO_2_max [25]. Probabilistic sensitivity analyses conducted by the authors showed that the probability that the intervention is cost-effective reached 56% based on the BMI outcome and 69% based on the VO2max outcome [25].

### Cost–utility studies

Three economic evaluations estimated cost-effectiveness results using the QALY framework alone [13, 27, 29]. One Australian study reported that a home-based self-managed exercise intervention was not cost-effective, with only a 5% probability that it was both less costly and more effective than the control [13]. The second study, from the Netherlands, found that a supervised group-based exercise intervention was not cost-effective compared with usual care with an ICER of €291,200 per QALY gained (US$375,572, 2016 prices) [27]. Scenario analysis, where the cost-effectiveness was considered from a healthcare perspective, provided similar to results of the baseline analysis [27]. The third study, also from the Netherlands, found that a home-based self-managed exercise intervention generated an ICER of €28,078 (US$35,707, 2016 prices) per QALY gained and the authors concluded that this was borderline cost-effective because the ICER of €28,078 fell below recommended cost-effectiveness thresholds [29]. Sensitivity analyses found that the outcomes were influenced by, first, utility values for the “menopausal symptoms” and “reduction in menopausal symptoms” health states and, second, the duration of the treatment effect, with shorter effect duration resulting in lower cost-effectiveness. The outcomes of this study were most sensitive to a reduction of the duration of the treatment effect from 5 to 3 and 1.5 years [29].

### Combined cost-effectiveness and cost–utility studies

Three studies estimated cost-effectiveness results using both QALY and non-QALY frameworks. An Australian study showed that a home-based self-managed and supervised physiotherapy intervention (ICER: AUS$ 1344 (US$1284) per QALY, 2016 prices) and a supervised group-based exercise and psychosocial intervention (ICER: AUS$ 14,478 (US$13,831) per QALY, 2016 prices) were both more effective than usual care, with the home-based intervention being the more cost-effective of the two experimental interventions [12]. In contrast, the results based on rehabilitated cases showed that usual care was less costly and more effective than both the home-based physiotherapy and group-based exercise and psychosocial interventions [12]. The ICERs for the two experimental interventions remained robust to several sensitivity analyses, with the exception of variations in utility scores to the lower limits of confidence intervals when QALYs were the outcome used. The authors conducted post hoc analyses to check whether self-reported function (FACT-B + 4) (used to estimate rehabilitated cases) and health utility scores (derived from Subjective Health Estimation scale) measured different concepts; they found that the measures were only modestly correlated (coefficient = 0.54, *p* < 0.001), which signified that the two outcome measures were sufficiently different and therefore different cost-effectiveness conclusions were possible given the study design [12].

Another Australian study reported that a home-based supervised exercise intervention was not cost-effective from either a health service provider (ICER: AUS$ 105,231 (US$73,786) per QALY, 2016 prices) or a private payer perspective (ICER: AUS$ 90,842 (US$63,697) per QALY, 2016 prices) [26]. In contrast, the authors reported that results based on rehabilitated cases showed that the home-based supervised exercise intervention was cost-effective, with an ICER of approximately AUS$2400 (US$1677, 2016 prices) per improver [26]. Sensitivity analyses indicated that the incremental cost-effectiveness ratios using QALYs gained were most sensitive to variations in EQ-5D-3L utility values within 95% confidence limits. Other variations in variables tested produced negligible changes to the incremental cost-effectiveness ratios. The likelihood of the service provider model being cost-effective was 44.4%, and 46.3% for the private model, at a cost-effectiveness threshold of AUS$50,000 per QALY gain. The authors concluded that whilst QALYs are the preferred measure of health consequence in health economic evaluations, there are a couple of reasons why they may not have been appropriate for this clinical context. Firstly, the intervention duration was not expected to extend participant survival during the trial period. Secondly, the mean health utility weight for the study participants (0.84) was similar to that reported for the Australian general population. Therefore, detecting differences in QALYs was deemed unrealistic in their sample [26].

A third study, conducted in the Netherlands, reported that a supervised exercise intervention was borderline cost-effective compared to usual care with an ICER of €26,916 (US$33,561, 2016 prices) per QALY gained [28]. The authors report that the non-QALY based results for this intervention suggest that it is cost-effective in terms of cost per unit change in general fatigue (ICER of €788), and cost per unit change in physical fatigue (ICER of €1402) [28]. The same study showed that a home-based self-managed exercise with the addition of behavioural reinforcement was not cost-effective compared with usual care with an ICER of €70,052 (US$87,347, 2016 prices) per QALY gained [28]. In contrast, the authors reported that home-based self-managed exercise with the addition of behavioural reinforcement is cost-effective in terms of cost per unit change in general fatigue (ICER of €4711), and cost per unit change in physical fatigue (ICER of €10,384) [28]. Scenario analyses conducted by the authors found that the probability of cost-effectiveness for both comparators was greater amongst compliant participants [28].

## Discussion

Evidence on the cost-effectiveness of exercise and physiotherapy interventions for the treatment of breast cancer was systematically assessed in this review. We identified only seven studies reporting on the cost-effectiveness of exercise and physiotherapy interventions for breast cancer patients [12, 13, 25–29], which between them evaluated nine different exercise-based interventions. These studies were generally of high quality and at low risk of bias. There have been two previous reviews that have reported evidence on the cost-effectiveness of exercise-based interventions in the treatment of breast cancer. The first review by Roine et al. [11] identified a single study, which reported that a home-based self-managed physiotherapy intervention and a supervised group-based exercise intervention with psychosocial support were more cost-effective than usual care [12]; this study is included in our review. The second review by Mewes et al. [10] also only identified a single study, which concluded that a home-based self-managed exercise intervention was not cost-effective compared to an active control consisting of flexibility and relaxation activities after breast cancer surgery [13]; this study is also included in our review.

Using QALYs as the primary measure of health consequence, the evidence surrounding the cost-effectiveness of exercise and physiotherapy interventions for breast cancer rehabilitation following surgery was equivocal. Three studies reported favourable cost-effectiveness results for different exercise or physiotherapy interventions [12, 28, 29]. In contrast, four studies conducted in different patient populations and healthcare settings found that exercise or physiotherapy interventions were not cost-effective using the QALY framework and on the basis of recommended country-specific cost-effectiveness thresholds for the QALY metric [13, 26–28].

Cost-effectiveness evidence was only reported within three countries, each with different healthcare systems (Australia, The Netherlands and France). This cost-effectiveness evidence was largely based on small studies with sample sizes ranging from 60 to 244 women. Methodological variations in recommended approaches across jurisdictions to the conduct of health economic evaluations may partly explain variations in cost-effectiveness results. For example, not all studies using the QALY framework for the analyses estimated QALYs using the same multi-attribute utility measure. The EQ-5D-3L was used in four studies [13, 26–28], whilst one study used utilities derived from the SHE [12] and a further study relied upon an external mapping algorithm [29]. Furthermore, variations in the content and delivery of exercise and physiotherapy interventions and the relative prices of the resource components of those interventions and their resource consequences are also likely to be factors driving the lack of consistency in findings. Consequently, any variation in cost-effectiveness estimates is likely to be driven, at least in part, by variations in methodological factors, as well as variations in the essential features of the interventions evaluated.

The comparators considered by the studies included in this systematic review can broadly be categorised as post-operative exercise versus control [12, 26], exercise versus control during adjuvant breast cancer treatment [13, 25, 27, 28] and exercise versus control following breast cancer treatment [29]. We found no economic evaluations comparing post-operative early versus delayed exercise interventions despite evidence for their clinical effectiveness [8]. Clearly, there is a need for further research that assesses the cost-effectiveness of the broad range of exercise and physiotherapy interventions that have been developed, many of which are used in routine clinical practice. A particular focus of future research should be to disentangle the contributing effects of frequency, intensity, time and type of exercise and physiotherapy interventions on cost-effectiveness outcomes with the view to specifying the relationship between features of those interventions and cost-effectiveness outcomes. Furthermore, although all but one study included in this systematic review measured health consequences in terms of QALYs, which are widely recommended for cost-effectiveness-based decision-making, there is a need for assessments of the sensitivity of widely used multi-attribute utility measures such as the EQ-5D-3L to changes in outcomes of interest, such as symptoms of fatigue [8].

The key strength of this study is the robust methodology adopted, which included following recommended guidelines for the conduct of systematic reviews of economic evaluations [18], and a transparent approach to study identification, assessment, data extraction and critical appraisal. Variations in methodological approaches and factors precluded the use of meta-analysis for combining cost-effectiveness evidence across studies, in line with other systematic reviews of economic evaluations [35, 36]. The study does have limitations, which should be borne in mind by readers. First, we did not search grey literature databases, including TRIP and Open Grey, within our search strategies. We worked closely with an information specialist to develop, test and refine our search strategies, but cannot preclude the possibility of exclusion of potentially relevant studies. Second, interpretation of the cost-effectiveness assessments that measured health consequences in terms of natural or biomedical units of outcomes, such as changes in BMI or cardiorespiratory fitness [25], is constrained by the absence of external cost-effectiveness thresholds for these health consequences. External evidence from stated or revealed preference studies on the value that should be placed on these effects will be required for the purposes of cost-effectiveness-based decision-making.

## Conclusion

This review has highlighted that the evidence base surrounding the cost-effectiveness of exercise and physiotherapy interventions for the treatment of breast cancer remains sparse with contrasting conclusions. Future research should particularly aim to broaden the evidence base by disentangling the contributing effects of frequency, intensity, time and type of exercise and physiotherapy interventions on cost-effectiveness outcomes.

## Electronic supplementary material

Below is the link to the electronic supplementary material.

Supplementary material 1 (DOCX 25 kb)

Supplementary material 2 (DOCX 25 kb)

Supplementary material 3 (DOCX 34 kb)
