# Supplementary material for: Systematic review of economic evaluations of exercise and physiotherapy for patients treated for breast cancer
Source: Breast Cancer Res Treat. 2019 Apr 17;176(1):37–52. doi: 10.1007/s10549-019-05235-7 (PMC6548756; doi:10.1007/s10549-019-05235-7)
Supplement: Supplementary file 1 — Supplementary material 1 (DOCX 25 kb) [file 10549_2019_5235_MOESM1_ESM.docx]

## Article title

Systematic review of economic evaluations of exercise and physiotherapy for patients treated for breast cancer

## Journal name

## Breast cancer research and treatment

## Authors

Mr Kamran Khan. Warwick Medical School, University of Warwick, UK

Mr Bruno Mazuquin. Warwick Medical School, University of Warwick, UK

Dr Alastair Canaway. Warwick Medical School, University of Warwick, UK

Prof Stavros Petrou. Warwick Medical School, University of Warwick, UK

Prof Julie Bruce. Warwick Medical School, University of Warwick, UK

## Corresponding author

Mr Kamran Khan [k.a.khan@warwick.ac.uk](mailto:k.a.khan@warwick.ac.uk).

# SEARCH STRATEGIES

# 1A): Search strategy used in MEDLINE & EMBASE

| 1 | exp Breast Neoplasms/ |
| --- | --- |
| 2 | (breast cancer$ or breast tumor$ or breast neoplasm$ or axillary dissection).tw. |
| 3 | (breast carcinoma$ or breast adenocarcinoma$ or breast sarcoma$).mp. |
| 4 | exp mastectomy/ |
| 5 | *Lymph node excision/ |
| 6 | (axill$ adj3 lymph node dissection).mp. |
| 7 | sentinel node dissection.mp. |
| 8 | Axillary clearance.tw. |
| 9 | Axillary node clearance.tw. |
| 10 | lymph node dissection.tw. |
| 11 | lumpectomy.tw. |
| 12 | or/1-11 |
| 13 | exp REHABILITATION/ |
| 14 | exp Physical Therapy Techniques/ or Physical Therapy Specialty/ |
| 15 | exp EXERCISE/ or exp EXERCISE THERAPY/ or exp EXERCISE MOVEMENT TECHNIQUES/ |
| 16 | exp Muscle Stretching Exercises/ |
| 17 | physical activity.mp. |
| 18 | exp Physical Conditioning, Human/ |
| 19 | (Physical education and training).af. |
| 20 | Physical Therapy Modalities/ |
| 21 | Musculoskeletal manipulations/ |
| 22 | (rehabilitat$ or physiotherap$ or manual therap$ or exercise$ or mobili$).mp. |
| 23 | exp Resistance Training/ |
| 24 | exp Self-Management/ |
| 25 | strength.mp. |
| 26 | or/13-25 |
| 27 | exp costs/ and cost analysis/ |
| 28 | cost-benefit analysis/ |
| 29 | quality-adjusted life years/ |
| 30 | economics,pharmaceutical/ |
| 31 | exp budgets/ |
| 32 | exp models, economic/ |
| 33 | exp decision theory/ |
| 34 | monte carlo method/ |
| 35 | markov chains/ |
| 36 | exp health status indicators/ |
| 37 | cost$.ti. |
| 38 | (cost$ adj2 (effective$ or utilit$ or benefit$ or minimis$)).ab. |
| 39 | economic$ model$.tw. |
| 40 | (economic$ or pharmacoeconomic$ or pharmaco-economic$).tw. |
| 41 | (price$ or pricing).tw. |
| 42 | (financial or finance or finances or financed).tw. |
| 43 | ((value adj2 money) or monetary).tw. |
| 44 | markov$.tw. |
| 45 | monte carlo.tw. |
| 46 | (decision$ adj2 (tree? or analy$ or model$)).tw. |
| 47 | (standard adj1 gamble).tw. |
| 48 | trade off.tw. |
| 49 | Health Status/ or "Quality of Life"/ or Quality-Adjusted Life Years/ or Health Status Indicators/ |
| 50 | or/27-49 |
| 51 | 12 and 26 and 50 |
| 52 | limit 51 to humans |

# 1B): Search strategy used in PubMed

| 1 | Search Breast Neoplasms [mh] |
| --- | --- |
| 2 | Search ((breast cancer*[tiab] OR breast tumor*[tiab] OR breast neoplasm*[tiab] OR axillary dissection[tiab])) |
| 3 | Search (breast carcinoma* or breast adenocarcinoma* or breast sarcoma*) |
| 4 | Search mastectomy [mh] |
| 5 | Search Lymph node excision [mh] |
| 6 | Search sentinel node dissection |
| 7 | Search Axillary clearance [tiab] |
| 8 | Search Axillary node clearance [tiab] |
| 9 | Search lymph node dissection [tiab] |
| 10 | Search lumpectomy [tiab] |
| 11 | OR/1-10 |
| 12 | Search REHABILITATION [mh] |
| 13 | Search (Physical Therapy Techniques [mh] or Physical Therapy Specialty [mh]) |
| 14 | Search (EXERCISE [mh] or EXERCISE THERAPY [mh] or EXERCISE MOVEMENT TECHNIQUES [mh]) |
| 15 | Search Muscle Stretching Exercises [mh] |
| 16 | Search physical activity [tw] |
| 17 | Search Physical Conditioning, Human [mh] |
| 18 | Search (Physical education and training) |
| 19 | Search Physical Therapy Modalities [mh] |
| 20 | Search Musculoskeletal manipulations [mh] |
| 21 | Search ((rehabilitat* or physiotherap* or manual therap* or exercise* or mobili*) |
| 22 | Search Resistance Training [mh] |
| 23 | Search Self-Management [mh] |
| 24 | Search strength |
| 25 | OR/12-24 |
| 26 | Search (costs [mh] and cost analysis[mh]) |
| 27 | Search cost-benefit analysis [mh] |
| 28 | Search quality-adjusted life years [mh] |
| 29 | Search economics,pharmaceutical [mh] |
| 30 | Search budgets [mh] |
| 31 | Search models, economic [mh] |
| 32 | Search decision theory [mh] |
| 33 | Search monte carlo method [mh] |
| 34 | Search markov chains [mh] |
| 35 | Search health status indicators [mh] |
| 36 | Search cost*[Title] |
| 37 | Search (cost* effective*[Title/Abstract] OR cost* utilit*[Title/Abstract] OR cost* benefit*[Title/Abstract] OR cost* minimis*[Title/Abstract] OR cost* consequenc*[Title/Abstract]) |
| 38 | Search economic* model* [tiab] |
| 39 | Search (economic* [tiab] or pharmacoeconomic* [tiab] or pharmaco-economic* [tiab]) |
| 40 | Search (price* [tiab] or pricing [tiab]) |
| 41 | Search (financial [tiab] or finance [tiab] or finances [tiab] or financed [tiab]) |
| 42 | Search markov* [tiab] |
| 43 | Search monte carlo [tiab] |
| 44 | Search (Health Status [mh] or "Quality of Life" [mh] or Quality-Adjusted Life Years [mh] or Health Status Indicators [mh]) |
| 45 | OR/26-44 |
| 46 | 11 and 25 and 45 |
| 47 | limit 46 to humans |

# 1C): Search strategy used in Web of Science

| 1 | TOPIC: (Breast Neoplasms) |
| --- | --- |
| 2 | TITLE: (breast cancer* or breast tumor* or breast neoplasm* or axillary dissection) |
| 3 | TITLE: (breast carcinoma* or breast adenocarcinoma* or breast sarcoma*) |
| 4 | TOPIC: (mastectomy) |
| 5 | TOPIC: (Lymph node excision) |
| 6 | TI=(axill* near/3 lymph node dissection) |
| 7 | TOPIC: (sentinel node dissection) |
| 8 | TITLE: (Axillary clearance) |
| 9 | TITLE: (Axillary node clearance or lymph node dissection or lumpectomy) |
| 10 | #9 OR #8 OR #7 OR #6 OR #5 OR #4 OR #3 OR #2 OR #1 |
| 11 | TOPIC: (REHABILITATION) |
| 12 | TOPIC: (Physical Therapy Techniques or Physical Therapy Specialty) |
| 13 | TOPIC: (EXERCISE or EXERCISE THERAPY or EXERCISE MOVEMENT TECHNIQUES) |
| 14 | TOPIC: (Muscle Stretching Exercises) |
| 15 | TITLE: (physical activity) |
| 16 | TOPIC: (Physical Conditioning, Human) |
| 17 | TITLE: (Physical education and training) |
| 18 | TOPIC: (Physical Therapy Modalities) |
| 19 | TOPIC: (Musculoskeletal manipulations) |
| 20 | TITLE: (rehabilitat* or physiotherap* or manual therap* or exercise* or mobili*) |
| 21 | TOPIC: (Resistance Training) |
| 22 | TOPIC: (Self-Management) |
| 23 | TOPIC: (strength) |
| 24 | #23 OR #22 OR #21 OR #20 OR #19 OR #18 OR #17 OR #16 OR #15 OR #14 OR #13 OR #12 OR #11 |
| 25 | TOPIC: (costs and cost analysis) |
| 26 | TOPIC: (cost-benefit analysis) |
| 27 | TOPIC: (quality-adjusted life years) |
| 28 | TOPIC: (economics,pharmaceutical) |
| 29 | TOPIC: (budgets) |
| 30 | TOPIC: (models, economic) |
| 31 | TOPIC: (decision theory) |
| 32 | TOPIC: (monte carlo method) |
| 33 | TOPIC: (markov chains) |
| 34 | TOPIC: (health status indicators) |
| 35 | TITLE: (cost*) |
| 36 | TI=(cost* near/2 effective* or utilit* or benefit* or minimis*) |
| 37 | TITLE: (economic* model*) |
| 38 | TITLE: (economic* or pharmacoeconomic* or pharmaco-economic*) |
| 39 | TOPIC: (price* or pricing) |
| 40 | TOPIC: (financial or finance or finances or financed) |
| 41 | TOPIC: (Health Status or "Quality of Life" or Quality-Adjusted Life Years) |
| 42 | #41 OR #40 OR #39 OR #38 OR #37 OR #36 OR #35 OR #34 OR #33 OR #32 OR #31 OR #30 OR #29 OR #28 OR #27 OR #26 OR #25 |
| 43 | #42 AND #24 AND #10 |

# 1D): Search strategy used in CINAHL

| S1 | MH Breast Neoplasms |
| --- | --- |
| S2 | TX breast cancer$ or breast tumor$ or breast neoplasm$ or axillary dissection |
| S3 | TX breast carcinoma$ or breast adenocarcinoma$ or breast sarcoma$ |
| S4 | (MH "Mastectomy+") |
| S5 | MH Lymph node excision |
| S6 | TX axill* N3 lymph node dissection |
| S7 | TX sentinel node dissection |
| S8 | TX Axillary clearance |
| S9 | TX Axillary node clearance |
| S10 | TX lymph node dissection |
| S11 | TX lumpectomy. |
| S12 | S1 OR S2 OR S3 OR S4 OR S5 OR S6 OR S7 OR S8 OR S9 OR S10 OR S11 |
| S13 | MH REHABILITATION |
| S14 | MW Physical Therapy Techniques or Physical Therapy Specialty |
| S15 | MW EXERCISE or EXERCISE THERAPY or EXERCISE MOVEMENT TECHNIQUES |
| S16 | MW Muscle Stretching Exercises |
| S17 | (MM "Physical Activity") |
| S18 | MW Physical Conditioning, Human |
| S19 | MW Physical education and training |
| S20 | MW Physical Therapy Modalities |
| S21 | MH Musculoskeletal manipulations |
| S22 | TX rehabilitat$ or physiotherap$ or manual therap$ or exercise$ or mobili$) |
| S23 | (MM "Resistance Training") |
| S24 | "Self-Management" |
| S25 | (MM "Muscle Strengthening+") OR (MM "Resistance Training") OR "strength" |
| S26 | S13 OR S14 OR S15 OR S16 OR S17 OR S18 OR S19 OR S20 OR S21 OR S22 OR S23 OR S24 OR S25 |
| S27 | MW costs and cost analysis |
| S28 | (MM "Cost Benefit Analysis") |
| S29 | (MM "Quality-Adjusted Life Years") OR (MM "Quality of Life+") OR (MM "Disability-Adjusted Life Years") |
| S30 | (MM "Economics, Pharmaceutical") |
| S31 | (MM "Budgets") |
| S32 | "models, economic" |
| S33 | "decision theory" |
| S34 | "monte carlo method" |
| S35 | "markov chains" |
| S36 | (MM "Health Status Indicators") |
| S37 | TI cost* |
| S38 | AB cost* N2 (effective * or utilit* or benefit* or minimis*) |
| S39 | TX economic* model* |
| S40 | TX economic$ or pharmacoeconomic$ or pharmaco-economic$ |
| S41 | TX price$ or pricing |
| S42 | TX financial or finance or finances or financed |
| S43 | TX value N2 (money or monetary) |
| S44 | TX markov$ |
| S45 | TX monte carlo |
| S46 | TX decision* N2 (tree or analy* or model*) |
| S47 | TX standard N1 gamble |
| S48 | TX time trade off |
| S49 | MH Health Status or "Quality of Life" or Quality-Adjusted Life Years |
| S50 | S27 OR S28 OR S29 OR S30 OR S31 OR S32 OR S33 OR S34 OR S35 OR S36 OR S37 OR S38 OR S39 OR S40 OR S41 OR S42 OR S43 OR S44 OR S45 OR S46 OR S47 OR S48 OR S49 |
| S51 | S12 AND S26 AND S50 |

# 1E): Search strategy used in PSYCHINFO

| 1 | exp Breast Neoplasms/ |
| --- | --- |
| 2 | (breast cancer$ or breast tumor$ or breast neoplasm$ or axillary dissection).tw. |
| 3 | (breast carcinoma$ or breast adenocarcinoma$ or breast sarcoma$).mp. |
| 4 | exp mastectomy/ |
| 5 | *Lymph node excision/ |
| 6 | (axill$ adj3 lymph node dissection).mp. |
| 7 | sentinel node dissection.mp. |
| 8 | Axillary clearance.tw. |
| 9 | Axillary node clearance.tw. |
| 10 | lymph node dissection.tw. |
| 11 | lumpectomy.tw. |
| 12 | 1 or 2 or 3 or 4 or 5 or 6 or 7 or 8 or 9 or 10 or 11 |
| 13 | exp REHABILITATION/ |
| 14 | exp Physical Therapy Techniques/ or Physical Therapy Specialty/ |
| 15 | exp EXERCISE/ or exp EXERCISE THERAPY/ or exp EXERCISE MOVEMENT TECHNIQUES/ |
| 16 | exp Muscle Stretching Exercises/ |
| 17 | physical activity.mp. |
| 18 | exp Physical Conditioning, Human/ |
| 19 | (Physical education and training).af. |
| 20 | Physical Therapy Modalities/ |
| 21 | Musculoskeletal manipulations/ |
| 22 | (rehabilitat$ or physiotherap$ or manual therap$ or exercise$ or mobili$).mp. |
| 23 | exp Resistance Training/ |
| 24 | exp Self-Management/ |
| 25 | strength.mp. |
| 26 | 13 or 14 or 15 or 16 or 17 or 18 or 19 or 20 or 21 or 22 or 23 or 24 or 25 |
| 27 | exp costs/ and cost analysis/ |
| 28 | cost-benefit analysis/ |
| 29 | quality-adjusted life years/ |
| 30 | economics,pharmaceutical/ |
| 31 | exp budgets/ |
| 32 | exp models, economic/ |
| 33 | exp decision theory/ |
| 34 | monte carlo method/ |
| 35 | markov chains/ |
| 36 | exp health status indicators/ |
| 37 | cost$.ti. |
| 38 | (cost$ adj2 (effective$ or utilit$ or benefit$ or minimis$)).ab. |
| 39 | economic$ model$.tw. |
| 40 | (economic$ or pharmacoeconomic$ or pharmaco-economic$).tw. |
| 41 | (price$ or pricing).tw. |
| 42 | (financial or finance or finances or financed).tw. |
| 43 | ((value adj2 money) or monetary).tw. |
| 44 | markov$.tw. |
| 45 | monte carlo.tw. |
| 46 | (decision$ adj2 (tree? or analy$ or model$)).tw. |
| 47 | (standard adj1 gamble).tw. |
| 48 | trade off.tw. |
| 49 | Health Status/ or "Quality of Life"/ or Quality-Adjusted Life Years/ or Health Status Indicators/ |
| 50 | 27 or 28 or 29 or 30 or 31 or 32 or 33 or 34 or 35 or 36 or 37 or 38 or 39 or 40 or 41 or 42 or 43 or 44 or 45 or 46 or 47 or 48 or 49 |
| 51 | 12 and 26 and 50 |
| 52 | limit 51 to humans |

# 1F): Search strategy used in The Cochrane Library

| 1 | MeSH descriptor: [Breast Neoplasms] explode all trees |
| --- | --- |
| 2 | (breast cancer$ or breast tumor$ or breast neoplasm$ or axillary dissection):ti,ab,kw (Word variations have been searched) |
| 3 | (breast carcinoma$ or breast adenocarcinoma$ or breast sarcoma$):ti,ab,kw (Word variations have been searched) |
| 4 | MeSH descriptor: [Mastectomy] explode all trees |
| 5 | MeSH descriptor: [Lymph Node Excision] explode all trees |
| 6 | (axill* near/3 lymph node dissection):ti,ab,kw (Word variations have been searched) |
| 7 | (sentinel node dissection):ti,ab,kw (Word variations have been searched) |
| 8 | (Axillary clearance):ti,ab,kw (Word variations have been searched) |
| 9 | (Axillary node clearance):ti,ab,kw (Word variations have been searched) |
| 10 | (lymph node dissection):ti,ab,kw (Word variations have been searched) |
| 11 | (lumpectomy):ti,ab,kw (Word variations have been searched) |
| 12 | OR 1-11 |
| 13 | MeSH descriptor: [Rehabilitation] explode all trees |
| 14 | MeSH descriptor: [Muscle Stretching Exercises] explode all trees |
| 15 | physical activity |
| 16 | exercise |
| 17 | MeSH descriptor: [Exercise] explode all trees |
| 18 | rehabilitat$ or physiotherap$ or manual therap$ or exercise$ or mobili$ |
| 19 | MeSH descriptor: [Resistance Training] this term only |
| 20 | MeSH descriptor: [Self-Management] explode all trees |
| 21 | strength |
| 22 | OR 13-21 |
| 23 | MeSH descriptor: [Costs and Cost Analysis] explode all trees |
| 24 | MeSH descriptor: [Cost-Benefit Analysis] explode all trees |
| 25 | MeSH descriptor: [Quality-Adjusted Life Years] explode all trees |
| 26 | MeSH descriptor: [Budgets] explode all trees |
| 27 | MeSH descriptor: [Models, Economic] explode all trees |
| 28 | MeSH descriptor: [Decision Theory] explode all trees |
| 29 | MeSH descriptor: [Monte Carlo Method] explode all trees |
| 30 | MeSH descriptor: [Markov Chains] explode all trees |
| 31 | MeSH descriptor: [Health Status Indicators] explode all trees |
| 32 | (cost$):ti,ab,kw |
| 33 | (cost$ near/2 (effective$ or utilit$ or benefit$ or minimis$)):ti,ab,kw |
| 34 | (economic$ model$):ti,ab,kw |
| 35 | (economic$ or pharmacoeconomic$ or pharmaco-economic$):ti,ab,kw |
| 36 | (price$ or pricing):ti,ab,kw |
| 37 | (financial or finance or finances or financed):ti,ab,kw |
| 38 | (value near/2 (money or monetary)):ti,ab,kw |
| 39 | (markov):ti,ab,kw |
| 40 | (monte carlo):ti,ab,kw |
| 41 | (decision$ near/2 (tree or analy$ or model$)):ti,ab,kw |
| 42 | standard near/1 gamble |
| 43 | trade off |
| 44 | MeSH descriptor: [Quality-Adjusted Life Years] explode all trees |
| 45 | OR 23-44 |
| 46 | 12 and 22 and 45 |

# 1G): Search strategy used in Scopus

| 1 | TITLE-ABS-KEY ( "Breast Neoplasms*"  OR  "breast cancer*"  OR  "breast tumor*"  OR  "axillary dissection"  OR  "breast carcinoma*"  OR  "breast adenocarcinoma*"  OR  "breast sarcoma*"  OR  mastectomy  OR  "Lymph node excision"  OR  "sentinel node dissection"  OR  "Axillary clearance"  OR  "Axillary node clearance"  OR  " lymph node dissection"  OR  lumpectomy ) |
| --- | --- |
| 2 | TITLE-ABS-KEY (REHABILITATION or "Physical Therapy Techniques" or "Physical Therapy Specialty" or EXERCISE or "EXERCISE THERAPY" or "EXERCISE MOVEMENT TECHNIQUES" or "Muscle Stretching Exercises" or "physical activity" or "rehabilitat*" or "physiotherap*" or "manual therap*" or "exercise*" or "mobili*" or "Resistance Training" or "Self-Management" or "strength") |
| 3 | TITLE-ABS-KEY ("costs" or "cost analysis" or "cost-benefit analysis" or "quality-adjusted life years" or "health status indicators" or "cost effective" or cost utility" or "cost minimis*" or "cost consequence*" or " Health Status" or "Quality of Life") |
| 4 | #1 AND #2 AND #3 |

# 1H): Search strategy used in EconLit

| 1 | Breast Neoplasms  OR breast cancer  OR  breast tumor OR  axillary dissection  OR  breast carcinoma OR  breast adenocarcinoma  OR  breast sarcoma  OR  mastectomy  OR  Lymph node excision  OR  sentinel node dissection  OR  Axillary clearance  OR  Axillary node clearance  OR   lymph node dissection  OR  lumpectomy |
| --- | --- |
| 2 | REHABILITATION or Physical Therapy Techniques or Physical Therapy Specialty or EXERCISE or EXERCISE THERAPY or EXERCISE MOVEMENT TECHNIQUES or Muscle Stretching Exercises or physical activity or rehabilitat* or physiotherap* or manual therap* or exercise* or mobili* or Resistance Training or Self-Management or strength |
| 3 | costs and cost analysis |
| 4 | cost benefit analysis |
| 5 | quality-adjusted life years |
| 6 | budget |
| 7 | SU models, economic |
| 8 | SU decision theory |
| 9 | SU monte carlo method |
| 10 | SU markov chains |
| 11 | SU health status indicators |
| 12 | TI cost* |
| 13 | cost effective* or cost utilit* or cost benefit* or cost minimis* or cost consequence* |
| 14 | economic* model* |
| 15 | Health Status or Quality of Life |
| 16 | S3 OR S4 OR S5 OR S6 OR S7 OR S8 OR S9 OR S10 OR S11 OR S12 OR S13 OR S14 OR S15 |
| 17 | S1 AND S2 AND S16 |
