# Supplementary material for: Systematic review of economic evaluations of exercise and physiotherapy for patients treated for breast cancer
Source: Breast Cancer Res Treat. 2019 Apr 17;176(1):37–52. doi: 10.1007/s10549-019-05235-7 (PMC6548756; doi:10.1007/s10549-019-05235-7)
Supplement: Supplementary file 2 — Supplementary material 2 (DOCX 25 kb) [file 10549_2019_5235_MOESM2_ESM.docx]

## Article title

Systematic review of economic evaluations of exercise and physiotherapy for patients treated for breast cancer

## Journal name

## Breast cancer research and treatment

## Authors

Mr Kamran Khan. Warwick Medical School, University of Warwick, UK

Mr Bruno Mazuquin. Warwick Medical School, University of Warwick, UK

Dr Alastair Canaway. Warwick Medical School, University of Warwick, UK

Prof Stavros Petrou. Warwick Medical School, University of Warwick, UK

Prof Julie Bruce. Warwick Medical School, University of Warwick, UK

## Corresponding author

Mr Kamran Khan [k.a.khan@warwick.ac.uk](mailto:k.a.khan@warwick.ac.uk).

# 2: CHEERS quality assessment of economic evaluation studies

| Study characteristics | Gordon 2005[1] | Gordon 2017[2]* | Haines 2010[3]* | May 2017[4]* | Mewes 2013[5] | Perrier 2016[6]* | Van Waart 2018[7]* |
| --- | --- | --- | --- | --- | --- | --- | --- |
| 1) Title | 1 | 1 | 1 | 1 | 1 | 1 | 1 |
| 2) Abstract | 1 | 1 | 1 | 1 | 1 | 1 | 1 |
| 3) Background and objectives | 1 | 1 | 1 | 1 | 1 | 1 | 1 |
| 4) Target population and subgroups | 1 | 1 | 1 | 1 | 1 | 1 | 1 |
| 5) Setting and location | 1 | 1 | 1 | 1 | 1 | 1 | 1 |
| 6) Study perspective | 1 | 1 | 1 | 1 | 1 | 1 | 1 |
| 7) Comparators | 1 | 1 | 1 | 1 | 1 | 1 | 1 |
| 8) Time Horizon | 1 | 1 | 1 | 1 | 1 | 1 | 1 |
| 9) Discount rate | 0 | 0 | 0 | 0 | 1 | 0 | 0 |
| 10 Choice of health outcomes | 1 | 1 | 1 | 1 | 1 | 1 | 1 |
| 11) Measurement of effectiveness | 1 | 1 | 1 | 1 | 1 | 1 | 1 |
| 12) Measurement of preference based outcomes | 1 | 1 | 1 | 1 | 1 | 0 | 1 |
| 13) Estimating Resource use and cost | 1 | 1 | 1 | 1 | 1 | 1 | 1 |
| 14) Currency, price date | 1 | 1 | 1 | 1 | 0 | 1 | 1 |
| 15) Choice of model | 1 | 0 | 0 | 0 | 1 | 0 | 0 |
| 16) Model Assumptions | 1 | 0 | 0 | 0 | 1 | 0 | 0 |
| 17) Analytic methods | 1 | 1 | 1 | 1 | 1 | 1 | 1 |
| 18) Study parameters | 1 | 1 | 1 | 1 | 1 | 1 | 1 |
| 19) Incremental cost and outcomes | 1 | 1 | 1 | 1 | 1 | 1 | 1 |
| 20) Uncertainty | 1 | 1 | 1 | 1 | 1 | 1 | 1 |
| 21) Heterogeneity | 0 | 0 | 0 | 0 | 0 | 0 | 0 |
| 22) Study findings, limitations | 1 | 1 | 1 | 1 | 1 | 1 | 1 |
| 23) Source of funding | 1 | 1 | 1 | 1 | 1 | 1 | 1 |
| 24) Conflicts of interest | 1 | 1 | 1 | 1 | 1 | 1 | 1 |
| Total | 22 | 20* | 20* | 20* | 22 | 19* | 20* |
| *These studies were based on trials with a time horizon of less than 12 months and 9, 15, and 16 did not apply to them. | | | | | | | |
