# Supplementary material for: Systematic review of economic evaluations of exercise and physiotherapy for patients treated for breast cancer
Source: Breast Cancer Res Treat. 2019 Apr 17;176(1):37–52. doi: 10.1007/s10549-019-05235-7 (PMC6548756; doi:10.1007/s10549-019-05235-7)
Supplement: Supplementary file 3 — Supplementary material 3 (DOCX 34 kb) [file 10549_2019_5235_MOESM3_ESM.docx]

## Article title

Systematic review of economic evaluations of exercise and physiotherapy for patients treated for breast cancer

## Journal name

## Breast cancer research and treatment

## Authors

Mr Kamran Khan. Warwick Medical School, University of Warwick, UK

Mr Bruno Mazuquin. Warwick Medical School, University of Warwick, UK

Dr Alastair Canaway. Warwick Medical School, University of Warwick, UK

Prof Stavros Petrou. Warwick Medical School, University of Warwick, UK

Prof Julie Bruce. Warwick Medical School, University of Warwick, UK

## Corresponding author

Mr Kamran Khan [k.a.khan@warwick.ac.uk](mailto:k.a.khan@warwick.ac.uk).

# 3A) Risk of bias - criteria

| **Judgement** | |
| --- | --- |
| **Domain** | **Support for judgement** |
| **Selection bias** | |
| Random Sequence generation | Describe the method used to generate the allocation sequence in sufficient detail to allow an assessment of whether it should produce comparable groups. |
| Allocation concealment | Describe the method used to conceal the allocation sequence in sufficient detail to determine whether intervention allocations could have been foreseen in advance of, or during, enrolment. |
| **Performance bias** | |
| Blinding of participants and personnel | Describe all measures used, if any, to blind study participants and personnel from knowledge of which intervention a participant received. Provide any information relating to whether the intended blinding was effective. |
| **Detection bias** | |
| Blinding of outcome assessment | Describe all measures used, if any, to blind outcome assessors from knowledge of which intervention a participant received. Provide any information relating to whether the intended blinding was effective. |
| **Attrition bias** | |
| Incomplete outcome data | Describe the completeness of outcome data for each main outcome, including attrition and exclusions from the analysis. State whether attrition and exclusions were reported, the numbers in each intervention group (compared with total randomized participants), reasons for attrition/exclusions where reported, and any re-inclusions in analyses performed by the review authors. |
| **Reporting bias** | |
| Selective reporting | State how the possibility of selective outcome reporting was examined by the review authors, and what was found. |
| **Other bias** | |
| Other source of bias | State any important concerns about bias not addressed in the other domains in the tool.  If particular questions/entries were pre-specified in the review’s protocol, responses should be provided for each question/entry. |

# 3B) Risk of bias - Summary

|  | Selection bias | | Performance bias | Detection bias | Attrition bias | Reporting bias | Other bias |
| --- | --- | --- | --- | --- | --- | --- | --- |
| Study | Random Sequence generation | Allocation concealment | Blinding of participants and personnel | Blinding of outcome assessment | Incomplete outcome data | Selective reporting | Other source of bias |
|  |  |  |  |  |  |  |  |
| Gordon, 2017[2] based on Hayes, 2013[8] | Low | Unclear | High | Low | Low | Low | Low |
| Haines, 2010[3] | Low | Low | High | Low | High | Unclear | Low |
| May, 2017[4]  Based on Travier, 2015[9] | Low | Low | High | Low | Low | Low | Low |
| Mewes, 2013[5]  Based on Duijts 2012[10] | Low | Unclear | High | Unclear | Low | Low | Low |
| Perrier, 2016[6]  Based on Touilaud, 2013[11] | Unclear | High | High | Unclear | Unclear | Unclear | Unclear |
| Van Waart, 2018[7]  Based on Van Waart, 2010[12] and Van Waart, 2015[13] | Low | Low | High | Unclear | Low | Low | Low |

# 3C) Risk of bias assessment Gordon 2017

| **Study:** Gordon, 2017 ( Hayes, 2013) | | |
| --- | --- | --- |
| **Domain** | **Review’s author judgement** | **Information supporting decision** |
| **Selection bias** | | |
| Random Sequence generation | Low | *“After baseline assessment, women were individually randomised into one of the three groups via a computer-generated, unblocked sequence of random numbers.”* |
| Allocation concealment | Unclear | No information of who allocated patients and how the generated numbers were concealed. |
| **Performance bias** | | |
| Blinding of participants and personnel | High | Due to the nature of the intervention it is not possible to blind therapists and patients. |
| **Detection bias** | | |
| Blinding of outcome assessment | Low | Disease and treatment characteristics were extracted from the Queensland Cancer Registry, including the type of cancer, type of surgery, tumour size, cancer stage and lymph node status, while a self- reported questionnaire and battery of physical tests were implemented by Exercise Physiologists blinded to group allocation. |
| **Attrition bias** | | |
| Incomplete outcome data | Low | Patients loss was low, only 14 out of 194. Intention-to-treat principles were applied to the analysis of data. No imputation was generated. |
| **Reporting bias** | | |
| Selective reporting | Low | All outcomes have been reported in Tables 3, 4 and 5. |
| **Other bias** | | |
| Other source of bias | Low | No concerns. |

# 3D) Risk of bias assessment Haines 2010

| **Study:** Haines, 2010 | | |
| --- | --- | --- |
| **Domain** | **Review’s author judgement** | **Information supporting decision** |
| **Selection bias** | | |
| Random Sequence generation | Low | *“Participants were randomized to intervention or control groups using a computer-generated randomization sequence that was entered into numbered, opaque, sealed envelopes by a study investigator (TH). The randomization sequence was held secure in an administration office separate from that of the investigators. Envelopes were only opened after completion of the initial assessment and intervention or control programs were provided to participants according to the allocation sequence following this.”* |
| Allocation concealment | Low | *“Participants were randomized to intervention or control groups using a computer-generated randomization sequence that was entered into numbered, opaque, sealed envelopes by a study investigator (TH). The randomization sequence was held secure in an administration office separate from that of the investigators. Envelopes were only opened after completion of the initial assessment and intervention or control programs were provided to participants according to the allocation sequence following this.”* |
| **Performance bias** | | |
| Blinding of participants and personnel | High | Impossible to blind, patients aware of what group they were allocated.  The intervention was based on a multimedia package, patients did not have contact with a therapist. |
| **Detection bias** | | |
| Blinding of outcome assessment | Low | *“Blinded baseline, 3, 6 and 12-month follow-up assessments.”* |
| **Attrition bias** | | |
| Incomplete outcome data | High | The intervention group had a 20% loss to follow-up, there is no information on intention-to-treat analysis or imputation.  Intervention= 9/46  Control= 7/43 |
| **Reporting bias** | | |
| Selective reporting | Unclear | Although table 3 brings a thorough description of the results, there is no information about the ROM results. |
| **Other bias** | | |
| Other source of bias | Low | No other concerns. |

# 3E) Risk of bias assessment May 2017

| **Study:** May, 2017 (Travier, 2015) | | |
| --- | --- | --- |
| **Domain** | **Review’s author judgement** | **Information supporting decision** |
| **Selection bias** |  |  |
| Random Sequence generation | Low | *“…they will be randomly allocated to the intervention or the control group by central data management.”* |
| Allocation concealment | Low | *“Allocation to the intervention- or control group will be concealed.”* |
| **Performance bias** |  |  |
| Blinding of participants and personnel | High | *“Because blinding of participants towards allocation is not feasible, this study has a pragmatic design.”* |
| **Detection bias** |  |  |
| Blinding of outcome assessment | Low | *“Blinding of participants was not possible due to the nature of the study, but outcome measures were assessed by researchers not involved with the participants.”* |
| **Attrition bias** |  |  |
| Incomplete outcome data | Low | Low loss to follow-up  Usual care=13/102  Intervention= 9/102 |
| **Reporting bias** |  |  |
| Selective reporting | Low | Results for all outcomes have been reported. Table 2 of Travier, 2015 and EQ-5D in May, 2017. |
| **Other bias** |  |  |
| Other source of bias | Low | No other concerns. |

# 3F) Risk of bias assessment Mewes 2015

| **Study:** Mewes, 2015 (Duijts, 2012) | | |
| --- | --- | --- |
| **Domain** | **Review’s author judgement** | **Information supporting decision** |
| **Selection bias** |  |  |
| Random Sequence generation | Low | *“…patients were randomly assigned to the CBT, PE, BT/PE, or control groups using computerized block randomization.”* |
| Allocation concealment | Unclear | No clear information whether the random numbers were concealed. |
| **Performance bias** |  |  |
| Blinding of participants and personnel | High | *“In view of the nature of the interventions, blinding of the participants and the researchers is not possible.”* |
| **Detection bias** |  |  |
| Blinding of outcome assessment | Unclear | There is no information whether the assessor was blinded to the intervention. |
| **Attrition bias** |  |  |
| Incomplete outcome data | Low | *“All analyses were conducted on an intention-to-treat (ITT) basis. In addition, per-protocol analyses were performed on patients who met criteria for minimal compliance with the intervention(s).”* |
| **Reporting bias** |  |  |
| Selective reporting | Low | Results were thoroughly described in tables 2 and table 3 in Duijts, 2012. |
| **Other bias** |  |  |
| Other source of bias | Low | No other concerns. |

# 3G) Risk of bias assessment Perrier 2016

| **Study:** Perrier, 2016 (Touilard, 2013) | | |
| --- | --- | --- |
| **Domain** | **Review’s author judgement** | **Information supporting decision** |
| **Selection bias** |  |  |
| Random Sequence generation | Unclear | No information about how patients were randomised and what method was used to generate the random numbers. |
| Allocation concealment | High | The trial was open-label |
| **Performance bias** |  |  |
| Blinding of participants and personnel | High | Not possible due to the nature of the intervention. |
| **Detection bias** |  |  |
| Blinding of outcome assessment | Unclear | No information provided. |
| **Attrition bias** |  |  |
| Incomplete outcome data | Unclear | No information provided. |
| **Reporting bias** |  |  |
| Selective reporting | Unclear | No information provided. |
| **Other bias** |  |  |
| Other source of bias | Unclear | Unable to judge other bias. |

# 3H) Risk of bias assessment Van Waart 2018

| **Study:** Van Waart, 2018 (Van Waart, 2015) | | |
| --- | --- | --- |
| **Domain** | **Review’s author judgement** | **Information supporting decision** |
| **Selection bias** |  |  |
| Random Sequence generation | Low | *“patients were randomly assigned to Onco-Move, OnTrack, or UC using the minimization method,^15^ which balanced groups with respect to age, primary diagnosis, treating hospital, and use of trastuzumab”* |
| Allocation concealment | Low | The minimization method was used to allocate patients after they agreed to take part in the study. |
| **Performance bias** |  |  |
| Blinding of participants and personnel | High | Blinding not possible due to the nature of the intervention |
| **Detection bias** |  |  |
| Blinding of outcome assessment | Unclear | No information provided whether assessor was blinded |
| **Attrition bias** |  |  |
| Incomplete outcome data | Low | Lost to follow-up  On Track = 5/76  Onco-Move= 8/77  Usual Care= 11/77  *“All analyses were conducted on an intention-to-treat basis.”* |
| **Reporting bias** |  |  |
| Selective reporting | Low | All outcomes have been reported. Tables 3 and 4 in Waart, 2015. |
| **Other bias** |  |  |
| Other source of bias | Low | No concerns. |

# References

1. Gordon, L.G., et al., *A cost-effectiveness analysis of two rehabilitation support services for women with breast cancer.* Breast Cancer Research & Treatment, 2005. **94**(2): p. 123-33.

2. Gordon, L.G., et al., *Cost-effectiveness of a pragmatic exercise intervention for women with breast cancer: results from a randomized controlled trial.* Psycho-Oncology, 2017. **26**(5): p. 649-655.

3. Haines, T.P., et al., *Multimodal exercise improves quality of life of women being treated for breast cancer, but at what cost? Randomized trial with economic evaluation.* Breast Cancer Research & Treatment, 2010. **124**(1): p. 163-75.

4. May, A.M., et al., *Cost-effectiveness analysis of an 18-week exercise programme for patients with breast and colon cancer undergoing adjuvant chemotherapy: the randomised PACT study.* BMJ Open, 2017. **7**(3): p. e012187.

5. Mewes, J.C., et al., *Cost-effectiveness of cognitive-behavioral therapy and physical exercise for alleviating treatment-induced menopausal symptoms in breast cancer patients.* Value in Health, 2013. **16 (3)**: p. A141.

6. Perrier, L., et al., *A cost-effectiveness analysis of a 6-month physical activity program versus usual dietary care during adjuvant chemotherapy in breast cancer patients.* Value in Health, 2016. **19 (3)**: p. A149.

7. van Waart, H., et al., *Cost–utility and cost-effectiveness of physical exercise during adjuvant chemotherapy.* European Journal of Health Economics, 2018. **19**(6): p. 893-904.

8. Hayes, S.C., et al., *Exercise for health: a randomized, controlled trial evaluating the impact of a pragmatic, translational exercise intervention on the quality of life, function and treatment-related side effects following breast cancer.* 2013. **137**(1): p. 175-186.

9. Travier, N., et al., *Effects of an 18-week exercise programme started early during breast cancer treatment: a randomised controlled trial.* 2015. **13**(1): p. 121.

10. Duijts, S.F., et al., *Efficacy of cognitive behavioral therapy and physical exercise in alleviating treatment-induced menopausal symptoms in patients with breast cancer: results of a randomized, controlled, multicenter trial.* 2012. **30**(33): p. 4124-4133.

11. Touillaud, M., et al., *Design of a randomised controlled trial of adapted physical activity during adjuvant treatment for localised breast cancer: the PASAPAS feasibility study.* 2013. **3**(10): p. e003855.

12. van Waart, H., et al., *Design of the Physical exercise during Adjuvant Chemotherapy Effectiveness Study (PACES): a randomized controlled trial to evaluate effectiveness and cost-effectiveness of physical exercise in improving physical fitness and reducing fatigue.* 2010. **10**(1): p. 673.

13. van Waart, H., et al., *Effect of low-intensity physical activity and moderate-to high-intensity physical exercise during adjuvant chemotherapy on physical fitness, fatigue, and chemotherapy completion rates: results of the PACES randomized clinical trial.* 2015. **33**(17): p. 1918-1927.
